# Raccoon Eye Caused by Dialysis-related Amyloidosis

**DOI:** 10.31662/jmaj.2024-0376

**Published:** 2025-03-28

**Authors:** Yuri Furuhashi, Tomotaka Takanosu, Masahiro Miki, Hiroko Kikuchi

**Affiliations:** 1 Emergency Medical Care Center, Ashikaga Red Cross Hospital, Ashikaga, Japan; 2 Emergency Medicine, Jichi Medical University, Shimotsuke, Japan

**Keywords:** raccoon eyes, dialysis-related amyloidosis, emergency medicine, primary care

A 64-year-old man undergoing hemodialysis for 17 years presented with periorbital ecchymosis without a history of head trauma (Figure 1). Laboratory tests and computed tomography showed no abnormalities, such as skull bone fractures. He has experienced a knee ligament injury and a trigger finger. Immunofixation electrophoresis detected no M-protein, and κ/λ ratio was within normal limits, thus immunoglobulin light-chain amyloidosis (AL amyloidosis) is excluded. Given his clinical manifestations, we clinically diagnosed dialysis-related amyloidosis (DRA). DRA is characterized by accumulation and tissue deposition of amyloid fibrils consisting of beta2-microglobulin in the bone, periarticular structures, and viscera. While periorbital purpura, or “raccoon eyes,” may appear in AL amyloidosis because of capillary fragility ^(1)^, cutaneous involvement in DRA is rare ^(2)^. Although biopsy is the gold standard for diagnosing DRA, it is rarely performed and is typically diagnosed clinically based on symptoms and imaging studies. Non-traumatic raccoon eyes can occur in dialysis patients, but it is essential to rule out AL amyloidosis.

**Figure 1.**
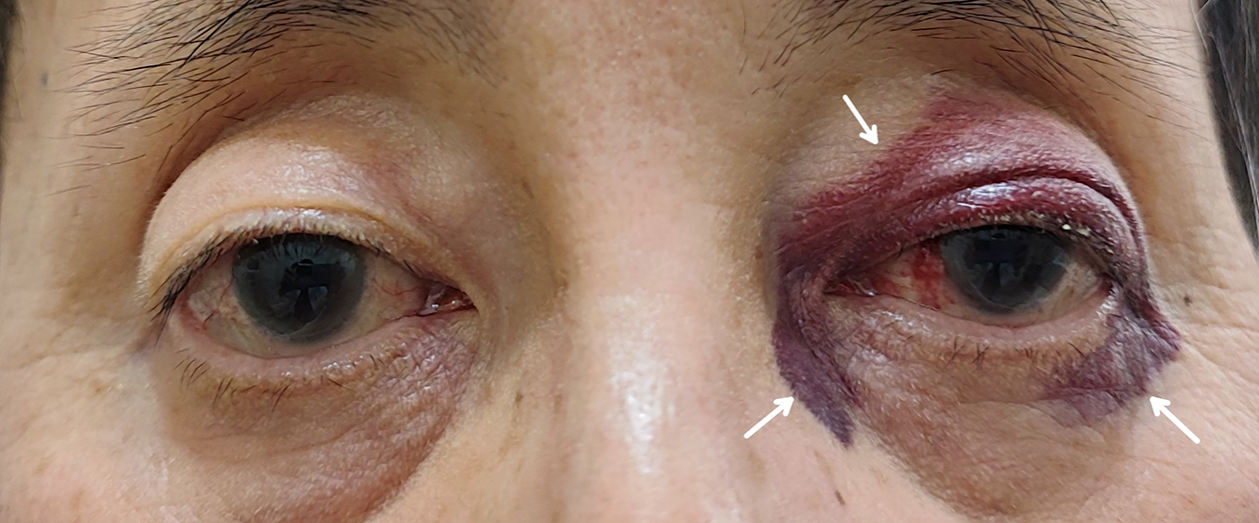
Left periorbital ecchymosis (white arrows) and subconjunctival hemorrhage.

## Article Information

### Conflicts of Interest

None

### Author Contributions

Y.F. wrote the manuscript. M.M. participated in the examination. T.T. edited the manuscript. H.K. gave clinical advice. All authors have reviewed the draft of the manuscript.

### Approval by Institutional Review Board (IRB)

IRB approval was not required in this study.

### Informed Consent

We obtained informed consent from the patient to publish his details.
